# Immediate Effects of Ultrasound-Guided Superior Gluteal Nerve-Targeted Manual Therapy and Exercise on Hip Abductor Strength, Gait Speed, and Pain in Early Postoperative Total Hip Arthroplasty: A Case Series

**DOI:** 10.7759/cureus.94708

**Published:** 2025-10-16

**Authors:** Mitsuaki Ashihara, Masashi Kawabata, Masato Aratake, Kazuma Miyatake

**Affiliations:** 1 Rehabilitation Center, Sagamihara Kyodo Hospital, Sagamihara, JPN; 2 Department of Rehabilitation, Kitasato University School of Allied Health Sciences, Sagamihara, JPN; 3 Department of Orthopedic Surgery, Sagamihara Kyodo Hospital, Sagamihara, JPN; 4 Department of Orthopaedic Surgery, Yokohama City University, Yokohama, JPN

**Keywords:** early rehabilitation, gait recovery, hip abductor strength, superior gluteal nerve, total hip arthroplasty, ultrasound-guided therapy

## Abstract

Hip abductor weakness is a common functional impairment in the early postoperative phase following total hip arthroplasty (THA), often contributing to gait disturbance, pain, and delayed recovery. Emerging evidence suggests that dysfunction of the superior gluteal nerve (SGN) may play a critical role in these impairments. However, clinical evidence for SGN-targeted rehabilitation remains limited. This case series aimed to explore the feasibility and preliminary effects of physiotherapy targeting the SGN on hip abductor strength, gait speed, and walking pain in the early postoperative period after THA.

Eight patients (three males and five females; aged 60-85 years) who underwent primary unilateral THA via the anterolateral approach were included. On postoperative day 14, all participants received ultrasound-guided manual therapy targeting the SGN combined with visual feedback-based therapeutic exercises for the gluteus medius and minimus. Outcome measures assessed immediately before and after the intervention included hip abductor strength measured with a handheld dynamometer, maximum gait speed measured with the 10-m walk test, and walking pain assessed with the Numerical Rating Scale (NRS).

Hip abductor strength improved from 3.4 ± 0.8 kgf to 5.3 ± 1.3 kgf. Gait speed increased from 0.55 ± 0.08 m/s to 0.73 ± 0.11 m/s. NRS pain scores for walking decreased from 3.6 ± 0.6 to 1.5 ± 0.7. Ultrasound assessment revealed consistent tenderness between the gluteus medius and minimus in all participants, supporting the rationale for SGN-focused intervention.

This case series demonstrated that a single session of ultrasound-guided manual therapy combined with targeted therapeutic exercise focusing on SGN function led to immediate improvements in hip abductor strength, gait speed, and walking pain in patients during the early postoperative phase after THA. These preliminary findings suggest the potential clinical value of incorporating nerve-focused interventions into standard rehabilitation programs following THA.

## Introduction

Total hip arthroplasty (THA) is a widely performed surgical procedure aimed at alleviating pain and restoring function in patients with end-stage osteoarthritis of the hip. Despite its overall success, postoperative rehabilitation is often challenged by significant weakness in the hip abductor muscles, particularly the gluteus medius and minimus. Muscle weakness is recognized as a major contributor to functional impairment and delayed recovery of ambulation and activities of daily living and is associated with increased pelvic instability, Trendelenburg gait, limping, and a higher risk of falls [1-3]. Therefore, restoring hip abductor function is a critical component of postoperative rehabilitation following THA.

Although hip abductor strength may recover to more than 80% of the contralateral side within 12 to 24 months postoperatively [4-6], it commonly decreases during the early postoperative period (one to three months). In particular, studies have indicated that one month after surgery, abductor strength may drop by up to 26% compared with preoperative levels [6,7]. The early postoperative period represents a crucial phase of functional recovery, during which physiological and structural changes profoundly influence outcomes.

In addition to surgical trauma to the abductor muscles, neurological factors have also been implicated in postoperative hip abductor weakness [8]. The superior gluteal nerve (SGN), which innervates the gluteus medius and minimus, is susceptible to intraoperative traction or compression injuries, potentially contributing to postoperative muscle weakness. Abitbol et al. reported that up to 77% of THA patients demonstrated subclinical SGN injury on electromyography [9]. Such nerve injuries may manifest clinically as Trendelenburg gait, decreased abductor strength, and compensatory external rotation of the hip. Because the SGN innervates muscles essential for gait and postural control, it should be considered a key therapeutic target in postoperative rehabilitation. Particularly in cases where muscle weakness may be driven by nerve dysfunction rather than disuse atrophy alone, appropriate physical therapy strategies should be emphasized.

Recently, ultrasound-guided hydrodissection targeting the SGN has received considerable attention as a potential therapeutic approach. This technique provides immediate and sustained improvements in both pain and muscle strength [10]. Ultrasound-guided interventions allow precise targeting of nerve- and fascia-related dysfunctions and have demonstrated efficacy in treating structural adhesions and impaired tissue gliding, such as in Achilles tendon adhesions [11]. Given this background, physical therapy interventions targeting the SGN may be effective for addressing early postoperative abductor muscle weakness following THA. However, clinical evidence supporting such interventions remains limited, and further development of novel treatment strategies is warranted.

This study examined the immediate effects of a physiotherapeutic intervention targeting the SGN on hip abductor strength and related functional outcomes in a case series of patients following THA, exploring the potential of this approach as a complement to conventional strengthening exercises in postoperative rehabilitation. We hypothesized that ultrasound-guided manual therapy combined with targeted exercise focusing on the SGN would produce immediate improvements in hip abductor strength, gait speed, and walking pain following THA.

## Materials and methods

Participants

The study included eight patients (three males and five females). The mean age was 72.4 years (range, 60-85 years), and the mean body mass index (BMI) was 24.8 kg/m² (range, 21.0-27.7 kg/m²). All patients underwent unilateral primary THA via the anterolateral approach. This retrospective observational study used preexisting clinical data. A passive refusal procedure was implemented through a public notice at the hospital, allowing patients to decline participation. Because all data were fully anonymized before analysis, the Institutional Review Board waived the requirement for written informed consent prior to the start of the study. The study was conducted in accordance with the Declaration of Helsinki and approved by the institutional ethics committee (approval number: 313).

Outcome measures

The intervention was performed on postoperative day 14, and assessments were conducted immediately before and after the physiotherapy intervention. The primary outcome was hip abductor muscle strength on the operated side. Secondary outcomes were maximum gait speed and pain during walking. Hip abductor strength was measured using a handheld dynamometer (µTas F-1, Anima Corp., Tokyo, Japan) following a previously established protocol [12]. Participants were positioned side-lying with the affected hip abducted to approximately 10°. The dynamometer was placed 5 cm proximal to the lateral femoral condyle. Participants were instructed to exert maximal effort for 5 s while lifting the leg upward, and the peak force from three trials was recorded. Maximum gait speed was assessed over a 16-m walkway, with the central 10 m timed and 3-m zones at both ends allocated for acceleration and deceleration [13]. Participants were allowed to use assistive devices, such as canes or walkers, as needed and were instructed to walk at the fastest safe pace. Pain during walking was assessed using an 11-point Numerical Rating Scale (NRS), ranging from 0 (no pain) to 10 (worst imaginable pain). Participants verbally rated their current pain level while walking. All outcome assessments were conducted by the same physical therapist who performed the intervention. Although blinding was not feasible, objective tools such as the handheld dynamometer, 10-m walk test, and NRS were used to minimize potential measurement bias.

Ultrasound-guided physical assessment and physical therapy

All participants continued standard postoperative physiotherapy, including range-of-motion and gait training, according to the hospital’s rehabilitation protocol. Analgesic medication use was managed by the attending orthopedic surgeons as part of routine postoperative care, and patients were allowed to use analgesics for pain as needed. The ultrasound-guided intervention was performed as an adjunct to routine rehabilitation. Postoperative day 14 was selected for the intervention because patients typically achieve adequate wound healing, pain control, and tolerance for active-assisted exercise at this stage. The safety of using ultrasound assessment and manual therapy around the surgical site was confirmed in consultation with the attending orthopedic surgeon before implementation.

**Figure 1:**
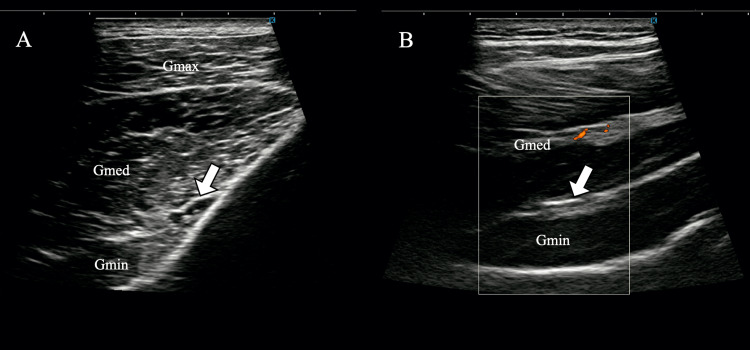
Tender points of the superior gluteal nerve identified under ultrasound guidance (A) Superior gluteal nerve near the suprapiriform foramen. (B) Inferior branch of the superior gluteal nerve, located between the gluteus medius and minimus. Arrows indicate the superior gluteal nerve. Gmax, gluteus maximus; Gmed: gluteus medius; Gmin: gluteus minimus.

Ultrasound imaging was used to assess tenderness in the region surrounding the SGN (Figure 1), revealing localized tenderness between the gluteus medius and minimus. Insufficient contraction of the gluteus minimus during hip abduction was also observed on ultrasonography (Video 1).

**Video 1:** Impaired contraction pattern of the gluteus minimus prior to intervention I: Iliac; Gmed: gluteus medius; Gmin: gluteus minimus.

**Figure 2:**
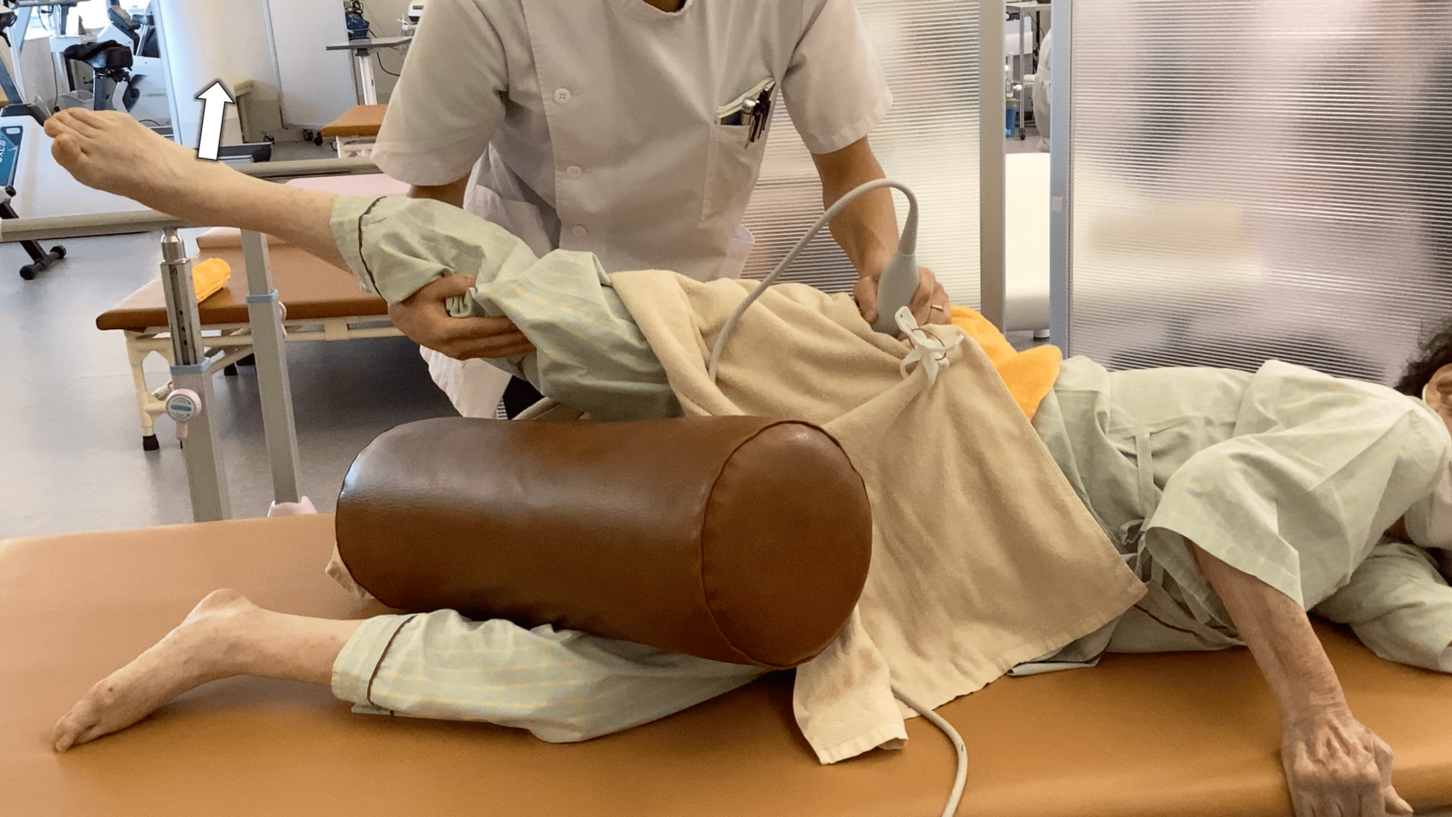
Active-assisted hip abduction exercise with real-time ultrasound feedback Patients were instructed to perform repeated contraction attempts during hip abduction until they could subjectively perceive the combined activation of the gluteus minimus and gluteus medius muscles.

The manual release technique was performed with moderate pressure sufficient to elicit mild tenderness and was maintained for approximately 3-5 minutes or until the tenderness subsided. The intervention was administered by a licensed physical therapist with more than 10 years of clinical experience in musculoskeletal rehabilitation. After manual release, patients performed contraction exercises for the gluteus minimus (Video 2) and active-assisted hip abduction exercises (Figure 2), with visual feedback provided by real-time ultrasound (Video 3). 

**Video 2:** Motor control exercises in a flexed and internally rotated hip position with real-time ultrasound feedback for selective gluteus minimus activation Gmed: gluteus medius; Gmin: gluteus minimus.

**Video 3:** Contraction of the gluteus minimus using visual feedback from the ultrasound imaging system I: Iliac; Gmed: gluteus medius; Gmin: gluteus minimus.

Statistical analysis

Continuous variables are presented as mean ± standard error (SE). Pre-post comparisons were performed with the Wilcoxon signed-rank test (two-tailed, α = 0.05). Effect size r was calculated and interpreted as small (0.10), medium (0.30), and large (0.50) [14]. All statistical analyses were performed using R (version 4.3.1; R Foundation for Statistical Computing, Vienna, Austria).

## Results

**Figure 3:**
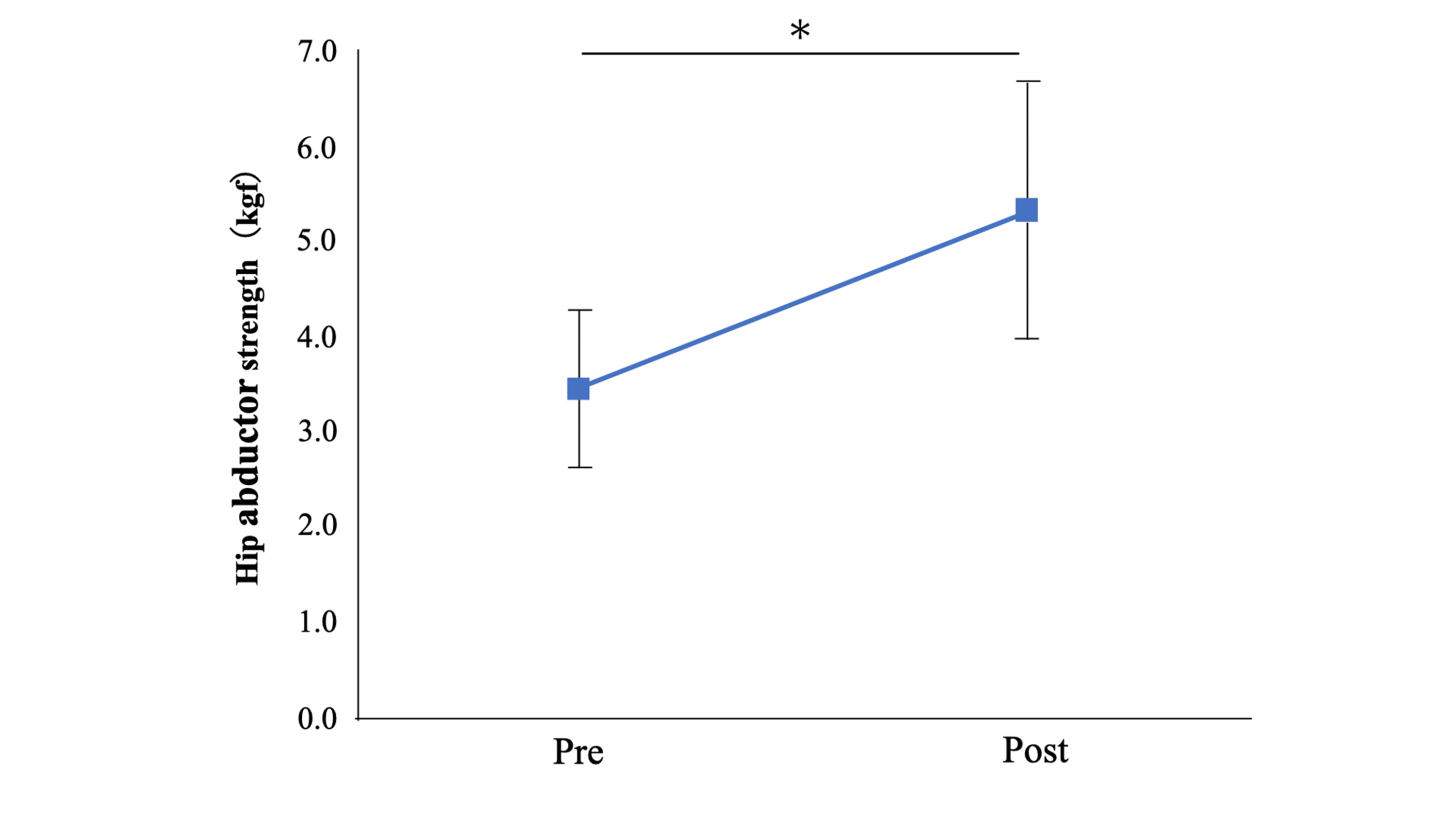
Changes in hip abductor muscle strength pre- and post-intervention Values are presented as mean, and error bars represent standard error. ＊ P<0.05

**Figure 4:**
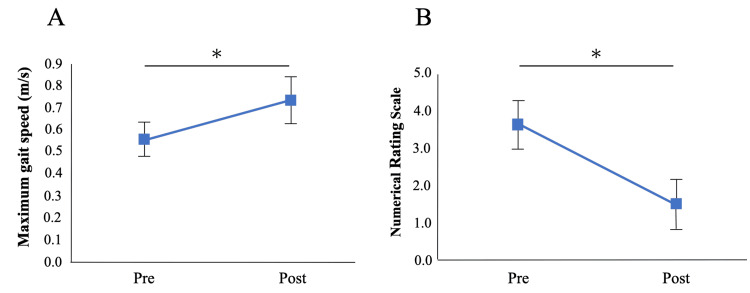
Changes in maximum walking speed and pain during walking pre- and post-intervention A: Maximum walking speed; B: Pain during walking Values are presented as mean, and error bars represent standard error. ＊ P<0.05

Individual patient characteristics and pre- and post-intervention outcome data are summarized in Table 1. Following the intervention, hip abductor muscle strength increased significantly from 3.4 ± 0.8 kgf to 5.3 ± 1.3 kgf (P = 0.02; Figure 3). The effect size (r = 0.86) indicated a large effect. Maximum gait speed also improved significantly, increasing from 0.55 ± 0.08 m/s to 0.73 ± 0.11 m/s (P = 0.01), with a large effect size (r = 0.86). In addition, pain during walking, as assessed by the NRS, decreased significantly from 3.6 ± 0.6 to 1.5 ± 0.7 (P = 0.008), accompanied by a large effect size (r = 0.91; Figure 4).

**Table 1:** Individual patient characteristics and pre- and post-intervention outcomes Age, sex, surgical side, BMI, and pre- and post-intervention values for hip abductor strength, gait speed, and walking pain (NRS) are shown. F: female; M: male; R: right; L: left; BMI: body mass index; NRS: numerical rating scale.

Patient	Age (years)	Sex	Surgical side	BMI (kg/m²)	Hip abductor strength (kgf)		Gait speed (m/s)		NRS pain score (0-10)	
					Pre	Post	Pre	Post	Pre	Post
1	60	F	L	25.4	4.5	7.0	0.5	0.8	3	1
2	80	M	L	24.1	1.9	3.8	0.5	0.6	7	5
3	72	F	R	27.7	1.2	2.9	0.5	0.9	6	4
4	74	F	L	21.2	1.8	2.1	0.5	0.5	2	0
5	71	F	R	25.2	2.9	3.8	0.4	0.5	3	0
6	85	M	L	27.2	5.2	7.0	0.9	1.2	2	0
7	77	F	R	21.0	1.8	1.8	0.2	0.3	4	2
8	60	M	L	26.3	8.5	14.2	0.9	1.1	2	0

## Discussion

In this case series, we observed immediate improvements in hip abductor strength, gait speed, and pain during walking two weeks after THA following the application of ultrasound-guided manual therapy targeting the SGN, combined with therapeutic exercises for the gluteus medius and minimus. These findings are novel in that they suggest the potential efficacy of a new therapeutic approach (manual intervention under ultrasound guidance directed at the SGN) as a means of addressing early postoperative abductor weakness. In contrast to conventional strengthening programs alone, this approach provides direct treatment for potential neural dysfunction and may offer clinically meaningful improvements in neuromuscular function and gait.

Ultrasound pressure-pain assessment frequently revealed tenderness between the gluteus medius and minimus. Anatomically, the SGN runs between these muscles and divides into superior and inferior branches. This region is susceptible to entrapment and adhesions caused by surrounding fascial and soft tissue structures [15]. Ultrasound-guided manual therapy targeting the SGN offers the advantage of high anatomical precision and enables selective and safe intervention. When ultrasound findings correlate with reproducible tenderness, targeted-release techniques may alleviate adhesions or compressions around the nerve, thereby reducing neuropathic pain and related symptoms and effects supported by prior reports [10]. In this study, it is plausible that manual release of the fascial plane between the gluteus medius and minimus contributed to the observed improvements in SGN-related buttock pain, hip abductor dysfunction, and gait pain.

Therapeutic exercises for the gluteus medius and minimus performed with real-time ultrasound visual feedback may enhance neuromuscular re-education and contribute to improvements in abductor strength. A systematic review by Valera-Calero et al. reported that motor control exercises incorporating ultrasound visual feedback increased muscle thickness, activation, and movement accuracy [16]. This method has been shown to be particularly effective for the deep trunk and hip muscles, as it allows patients to visualize contractions during movement and facilitates more precise motor pattern retraining. Compared with verbal or tactile feedback, ultrasound feedback has consistently demonstrated superior effectiveness across multiple studies. In the present study, patients were able to visually recognize contractions of the gluteus minimus, which is noteworthy. Such visualization-based feedback strategies enhance proprioceptive awareness and facilitate effective motor learning. The gluteus minimus has been shown to be highly active during single-leg stance and walking, as demonstrated in magnetic resonance imaging and fluorodeoxyglucose positron emission tomography studies [17,18]. In this study, patients’ ability to voluntarily activate the gluteus minimus may have improved pelvic stability during gait, thereby contributing to the observed increase in walking speed.

This study had several limitations. First, the small sample size of eight patients limits the generalizability of the findings. Second, the absence of a control group precludes definitive conclusions regarding causal relationships between the intervention and the observed outcomes. Therefore, the improvements should be interpreted as associations rather than evidence of direct causality. Further controlled studies are needed to confirm whether SGN-targeted interventions directly lead to improvements in hip abductor strength, gait performance, and pain reduction. Additionally, this study was not blinded, as the same therapist performed both the intervention and the outcome assessments, which may introduce observer bias. Moreover, the manual release technique was not fully standardized or quantified in terms of pressure or duration, potentially limiting reproducibility across practitioners. Third, only short-term effects were evaluated; therefore, the medium- and long-term sustainability of the intervention remains unknown. Future studies should incorporate neurophysiological assessments such as electromyography and advanced imaging techniques to clarify the mechanisms underlying the observed effects and evaluate the long-term efficacy of the intervention.

## Conclusions

This exploratory case series suggests that ultrasound-guided manual therapy targeting the SGN, combined with gluteus medius and minimus exercise therapy, is associated with immediate improvements in hip abductor strength, gait speed, and pain during walking in the early postoperative phase following THA. These preliminary findings indicate a potential role for nerve-focused physiotherapeutic strategies in enhancing functional recovery; however, controlled studies are needed to confirm their efficacy and long-term benefits.
